# Health & Wealth: is weight loss success related to monetary savings in U.S. adults of low-income? Findings from a National Study

**DOI:** 10.1186/s12889-019-7711-3

**Published:** 2019-11-21

**Authors:** Kerem Shuval, Bob M. Fennis, Qing Li, Amir Grinstein, Meike Morren, Jeffrey Drope

**Affiliations:** 1 Economic and Health Policy Research Program, Department of Intramural Research, American Cancer Society, Atlanta, GA USA; 2 Faculty of Social Welfare & Health Sciences, School of Public Health, University of Haifa, Haifa, Israel; 3 Department of Marketing, University of Groningen, Groningen, The Netherlands; 4 School of Marketing and International Business, Victoria University of Wellington, Wellington, New Zealand; 5 D’Amore‐McKim School of Business, Northeastern University, Boston, MA USA; 6 Faculty of Economics and Business Administration, Vrije Universiteit Amsterdam, Amsterdam, The Netherlands

**Keywords:** Weight loss, Monetary savings, Self-regulation

## Abstract

**Background:**

Many individuals aspire to attain various goals in life, such as committing to a healthful diet to slim down or saving for retirement to enhance future welfare. While these behaviors (weight loss and saving) share the common denominator of self-regulation, it is unclear whether success in one domain is related to the other. Therefore, we examined the relationship between long term weight loss (LTWL) success and monetary savings among U.S. adults who at one point in life diverged from normal weight status.

**Methods:**

Data on 1994 adults with a maximum BMI ≥ 25 kg/m^2^ and with an annual household income equal or less than 200% poverty level. Data were derived from a U.S. population-based study (NHANES). The independent variable was LTWL success (loss maintained for at least 1 year), which was operationalized as < 10% (reference group), 10.00–19.99%, and ≥ 20.00%. The dependent variable was monetary savings (e.g., 401 K), defined as a 3-category ordinal variable. We employed ordered logistic regression to estimate the relationship between LTWL success and increased odds for higher overall savings.

**Results:**

Multivariable analysis revealed that adjusting for income, education and other covariates, being in the highest LTWL category (≥20.00%) significantly reduced the likelihood of monetary savings in comparison to the reference group (OR = 0.55, 95%CI = 0.34–0.91). This relationship was not observed in the lower LTWL category (10.00–19.99%).

**Conclusions:**

Adults who in the past were overweight or obese and who presently exhibit high levels of LTWL, were markedly less successful when it came to their finances. This might stem from significant cognitive-affective resources exerted during the weight loss process coupled with a paucity of financial resources which impede financial decision making. This supposition, however, warrants future research.

## Background

Despite New Year’s resolutions to exercise and eat well to lose excess weight, most individuals are not able to follow through with their plan despite their best intentions [1]. In fact, only ~ 20% of adults who are overweight successfully lose weight [2]. Indeed, more than two thirds of American adults are overweight or obese, which is mostly due to human behavior and environment, not genetics [3, 4]. This situation incurs increased morbidity and soaring health care costs [5, 6]. Similarly, while individuals are cognizant of the importance of saving for retirement, actual savings rates in the U.S. represent only ~ 5% of disposable income [7]. Moreover, the median retirement savings account balance for the working-age American household is $3000, and remains alarmingly low ($12,000) even for those nearing retirement age [8].

Both health and wealth outcomes require a self-regulatory effort in the form of goal setting, planning, overcoming hurdles and resisting immediate impulses [9, 10]. Goal setting (e.g., losing weight) and controlling emotions is required to preempt immediate gratification urges when temptation arises [9, 10]. For example, when primed to eat chocolate cake an individual with a weight loss goal who has a high degree of self-regulation will likely opt for an apple rather than eating the cake to achieve their goal [11, 12].. In the psychological literature, while both constructs are frequently used interchangeably, self-regulation generally refers to intentional and deliberate actions by which individuals plan, monitor and alter their cognitions, emotions, and behaviors in the service of long term goals [13, 14]. Self-control, more specifically, refers to suppressing, overcoming or channeling acute impulses to the extent that they interfere with valued long term goals [14].

In economics, health and wealth decisions are viewed as inter-temporal decisions between myopic and far-sighted choices [15]. That is, individuals with ‘near sighted’ (impatient) preferences will devalue future rewards (known as delayed discounting), such as future health benefits, over present day immediately gratifying choices (e.g., eating ice-cream) [11, 16–18]. Devaluing greater future benefits often occurs since the future rewards are not salient at present [19]. Individuals able to delay immediate gratifying behavior are regarded as having patient time preferences, which has been linked in the literature to less impulsive behavior [20]. Whereas traditional micro-economic theory regarded time preference as stable over time, more recent modeling integrates insights from psychology by acknowledging that time preferences could change over time, particularly when in a visceral state (e.g., when angry or hungry) [21–23]. For example, if one’s goal is to adhere to a healthful diet yet social and environmental cues prime them to consume energy dense foods, their a priori goal will often not be met. This ‘scenario’, where the a priori goal is not met, is regarded as an inconsistent time preference and reflects a self-control problem [24].

Both fields (psychology and economics) concur that the ability to prioritize ‘should’ (e.g., walking on the treadmill) over ‘want’ (e.g., sitting on the couch watching TV) behaviors to achieve higher levels goals (e.g., chronic disease prevention), is indicative of higher levels of self-control or self-regulation [25, 26]. Henceforth, in the current study we use the term ‘self-regulation’. High self-regulation has been linked to behaviors that enhance health and well-being, such as educational attainment, monetary savings, and obesity prevention [27–30]. In the health domain, for example, a study by Stoklosa et al. (2018), among a national sample of U.S. adults, observed that higher self-regulation is linked to reduced obesity levels among adults and their children [21]. Moreover, Fan and Jin (2014) found that individuals who are obese exhibit lower levels of self-regulation than their normal weight counterparts, and that lower self-regulation is associated with unhealthful eating and physical inactivity [31]. In the specific case of self-regulation in financial decision making, research by Gubler and Pierce (2014) observed that employees who saved for retirement exhibited better blood test scores [32]. Similarly, Israel et al. (2014) found that higher credit scores, arguably indicative of financial prudence, were predictive of lower cardiovascular disease risk, irrespective of income [33].

The focal point of these studies, however, was on individuals with high self-regulation rather than the cross-domain behavior of those with lower levels. Furthermore, these studies have insufficiently focused on low-income individuals at increased risk for obesity and other chronic diseases, with less financial resources, and less access to medical care [34]. To this end, the current study focuses on weight loss and financial behaviors of low-income adults who were historically overweight. That is, at one point in life participants ceased maintaining normal weight status (henceforth, historically overweight). Thus, the present study aims to describe the relationship between long term weight loss (LTWL) success and monetary savings among adults who have been historically overweigh.

## Methods

### Data and participants

Data were derived from the National Health and Nutrition Examination Survey (NHANES); described elsewhere [35]. Briefly, NHANES is conducted on an ongoing basis bi-annually; the study design is cross-sectional, and includes information on health, nutrition, and weight history [36]. In the current study we utilize 4 survey waves: 2007–8, 2009–10, 2011–12, 2013–14. These waves represent observations at one-time point; that is, they are not longitudinal in nature. A total of 7172 adults aged 22–59 years who were not underweight (BMI < 18.5 kg/m^2^), not pregnant and not told by their physician that they have a heart disease or stroke were considered for inclusion after meeting the following criteria: historically overweight (i.e., maximum BMI of ≥25 kg/m^2^), [37] able to work, with no physical/mental limitations, or cognitive impairment. Of these, 5178 observations were omitted due to incomplete information on the primary independent variable (LTWL), dependent variable (monetary savings), and covariates (as described below). This resulted in an analytic dataset of 1994 participants.

This study sample consisted of low-income participants since only those reporting a family annual income of equal or less than 200% poverty level were asked to respond to questions pertaining to their monetary savings [36]. Thus, the present study sample (*n* = 1994) differs from the omitted observations (*n* = 5178) both in socio-demographic characteristics and health status. For example, in comparison to the omitted sample not meeting inclusion criteria, the present study sample had a markedly lower income, (e.g., 31.6% vs. 4.0% had an annual household income of less than $20,000), was less educated (14.4% vs. 37.2% with a college education), and fewer reported being in excellent health (8.4% vs. 12.2%). Hence the present study sample is not representative of all NHANES participants. In addition, it should be noted that NHANES obtained informed consent from participants and received ethics approval from the National Center for Health Statistics Research Ethics Review Board. The current study received exempt status from the Morehouse School of Medicine Institutional Review Board.

### Primary independent and dependent variable

The primary independent variable was LTWL, which was based on reported maximum weight, weight 1 year ago, and the present weight [37]. More specifically, LTWL (i.e., loss maintained for at least 1 year) was computed by subtracting either the current or the weight 1 year ago (the highest of the two) from participants’ maximum weight. This was then divided by the current weight and multiplied by 100. This is based on the approach taken by Kraschnewski (2010) and Knell (2018) [37, 38]. The LTWL percentage was grouped into the following 5 categories in adherence with previous research: 0.0–4.9% (reference group), 5.0–9.9%, 10.0–14.9%, 15.0–19.9%, and ≥ 20.0% [37, 38]. To avoid sparse cells, [39] we then grouped this variable into 3 clinically meaningful categories: 0.0–9.9% (reference group), 10.0–19.9%, and ≥ 20.0%. The 10% cutoff was selected since the evidence indicates that losing 10% or more of one’s maximum body weight (and maintaining it over time) is related with a significant reduction in chronic morbidity risk [2, 37, 40]. Grouping this variable into 5 or 3 categories did not change results materially in multivariable analysis.

The primary dependent variable was total savings, an ordinal variable based on two questions. In the first question, participants reported if they (or a household member) currently have more than $5000 in savings in all accounts including cash, savings/checking, stock, mutual funds, and retirement (e.g., 401 K) [36]. If they responded affirmatively then they were not asked the second question. If they indicated less than $5000 in savings, they were asked to select one of the following categories: <$500, $501–$1000, $1001–$2000, $2001–$3000, $3001–$4000, and $4001–$5000. From these two questions a 3-category saving variable was created (<$500, $501–$5000, >$5000). We utilized a 3 group categorization for the saving variable rather than the original categories to avoid sparse cells [39]; though results did not change materially using either approach.

### Covariates

The following variables were considered as potential confounders in line with previous research: [37, 38] college graduate (yes/no), annual household income (<$20,000; $20,000–$44,999, $45,000–$74,999, ≥$75,000), and having a diet goal (yes/no). That is, participants who responded positively to one or more of the following three questions were regarded as having a diet goal: (1) Was their weight change in the past year intentional; (2) Did they try to lose weight in the past year; and (3) Did they take action to prevent weight gain in the past year. Conversely, those responding negatively to all three questions were defined as not having a diet goal. Additionally, the following covariates were adjusted for the analysis: self-reported health status (fair, good, very good, excellent), current smoking (3.08 ng/mL cotinine cutoff), [41] gender, age, race/ethnicity (Hispanic, non-Hispanic black, non-Hispanic white, other), marital status (married, divorced/separated, widowed, never married), and household size.

### Statistical analysis

The relationship between LTWL (primary independent variable), to monetary savings (dependent variable) was estimated using multivariable ordered logistic regression while adjusting for covariates (age, race/ethnicity, gender, annual household income, household size, marital status, college education, self-reported health status, current smoking, and having a diet goal) based on prior research [37, 38]. Ordered regression was utilized due to the ordinal nature of the saving variable, where higher categories indicate increased odds for more savings [42, 43]. Results from the model are presented as adjusted odds ratios (OR) and their 95% confidence intervals (CI) for a transition to a higher saving group versus remaining in the same category. Due to the complex multistage random sampling of NHANES, weight procedures were used [44]. More specifically, the mobile exam weights (provided by NHANES) of each of the survey waves (2007–9, 2009–10, 2011–12, 2013–14) were combined and divided by 4 to represent 8 year weights [44]. STATA 13.1 (Stata-Corp LP) was used in all statistical analyses. The threshold for statistical significance was set at α < 0.05.

## Results

The socio-demographic characteristics (weighted) of the study sample are presented in Table 1. Briefly, the mean age of participants was 36.5 years (SE = 0.35), and 48.6% were women. Slightly less than half (48.5%) were non-Hispanic whites, 28.4% were Hispanic, and 17.4% were non-Hispanic black. Furthermore, 14.4% were college graduates, and 84.6% had an annual household income of less than $45,000.

**Table 1:** Descriptive Characteristics (Weighted) of Sample: NHANES 2007–2014 (*n* = 1994)

Characteristics	Weighted %	SE
Gender		
Women	48.65%	0.01
Age (years): mean (SE)	36.50	0.35
Race/Ethnicity		
Non-Hispanic White	48.54%	0.03
Non-Hispanic Black	17.38%	0.02
Hispanic	28.41%	0.02
Other	5.67%	0.01
Marital Status		
Married	43.32%	0.02
Widow	0.91%	0.00
Divorced/Separated	16.07%	0.01
Never married	25.69%	0.02
College Graduate	14.45%	0.01
Household Size: mean (SE)	3.803	0.05
Annual Household Income (U.S. Dollars)- categories		
< $20,000.00	31.62%	0.01
$20,000.00–$44,999.00	53.00%	0.01
$45,000.00–$74,999.00	9.78%	0.01
≥ $75,000.00	5.60%	0.01
Long-term Weight Loss Maintenance (0.00–9.99%)	71.85%	0.01
Long-term Weight Loss Maintenance (10.00–19.99%)	20.34%	0.01
Long-term Weight Loss Maintenance (≥20.00%)	7.81%	0.01
Self-reported Health Status		
Excellent	8.47%	0.01
Very good	27.74%	0.01
Good	44.73%	0.01
Fair	17.33%	0.01
Poor	1.72%	0.00
Current Smoker	40.30%	0.02
Diet Goal	56.44%	0.01
Total Savings (U.S. Dollars)		
< $500.00	72.61%	0.02
$501.00–$5000.00	13.12%	0.01
> $5000.00	14.28%	0.02

*NHANES* National Health and Nutrition Examination Survey, *SE* standard error, NHANES weights were utilized

Multivariable analysis, presented in Table 2, reveals that in comparison to the reference group (< 10% LTWL), a significant inverse relationship was found between the ≥20% LTWL category and total savings. That is, individuals in the highest LTWL category were 45% less likely to save (95%CI = 0.34–0.91) than the reference group. This relationship was not observed for the lower, 10–19.99% LTWL category (OR = 0.92; 95%CI 0.69–1.23). In addition, having a diet goal, college graduation, and a higher annual income were each independently and positively related to increased savings. Specifically, participants with a diet goal were 1.46 times more likely to save (95%CI 1.13–1.90), college graduates were 2.95 times more likely to save (95%CI 1.89–4.60), and those in the highest income strata were 6.22 times more likely to save (95%CI 2.84–13.61).

**Table 2:** Long-term Weight Loss and Monetary Savings: ordered logistic regression^a^

	Total Savings^a^ OR 95%CI
Long-term Weight Loss-categories (< 10% reference)	
10.00–19.99% Weight Loss	0.92 0.69–1.23
≥ 20.00% Weight Loss	0.55* 0.34–0.91
Diet Goal	1.46* 1.13–1.90
College Graduate	2.95* 1.89–4.60
Annual Household Income (<$20,000 reference)	
$20,000.00–$44,999.00	1.42 0.99–2.12
$45,000.00–$74,999.00	3.04* 1.88–4.91
$ ≥ 75,000.00	6.22* 2.84–13.61

*CI* confidence intervals, *OR* odds ratio. An *indicates statistical significance at the *p* < 0.05 level

^a^The multivariable regression model adjusted for age, race/ethnicity, gender, household size, marital status, self-reported health status, and current smoking; NHANES weights were utilized. Ordered logistic regression was used due to the ordinal nature of the dependent variable

## Discussion

The inverse relationship observed between LTWL and monetary savings might stem from the fact that in this study sample participants were historically overweight, which could indicate lower overall self-regulation [45, 46]. Thus, unlike individuals with high self-regulation who are able to control their thoughts, goals and behaviors across domains, [47] those with lower levels might be able to exert self-control in some domains but not in others. This finding might be explained by the significant cognitive-affective effort exerted by the group in the process of losing 20% (or more) of their maximum weight, [12] which did not leave enough “mental resources” deployed when it came to financial decision-making. This might also be compounded by the fact that low-income participants experience a preponderance of adverse life events, which assail mental resources and detrimentally affect decision-making [48–50]. Theses suppositions are preliminary, however, and should be substantiated in study samples which contain additional information pertaining to adverse life events, psychological mechanisms, social and environmental variables, as well as more elaborate data pertaining to spending and saving behaviors.

Nonetheless, these findings are novel and important since they illustrate how decisions in different domains are interrelated. These decisions, namely to lose weight and save money, have paramount health and welfare implications. The field of public health has traditionally focused on social determinants of health, that is, how one’s socio-demographic and economic status affects health [51]. Evidence clearly points to the fact that individuals with low-incomes have higher prevalence rates of obesity, type 2 diabetes, cardiovascular diseases, and premature death, while having less access to quality medical care in comparison to their higher income counterparts [52–54]. The increased risk for chronic morbidity and mortality among individuals with low incomes likely stems from a higher rate of ‘behavioral risk factors’, such as physical inactivity and unhealthful eating leading to obesity, which is due to living in environments conducive to these behaviors [55]. Research, however, has rarely assessed whether health behaviors are related to monetary savings, both sharing a common underlying factor of self-regulation.

It should be noted that as with the Gubler and Pierce (2014) study, a direct measure of self-regulation was not available in the present study. However, analysis reveals that individuals possessing a diet goal were significantly more likely to save money. While a diet goal leads to goal directed action in the eating domain, [12] it also might be related to achieving higher level goals in other domains. Hence, having a diet goal might be a proxy for higher overall self-regulation versus lower self-regulation among those without a diet goal in this sample [35, 41–43]. This assumption, however, needs to be substantiated in future research. Additionally, the NHANES dataset is cross-sectional, which precludes determining a temporal relationship. Moreover, whereas LTWL is based on an individual’s weight at three time points, the exact timeframe in which the financial savings occurred is not clear. Further, the monetary saving behavior was based on either individual or household savings, which does not necessarily reflect an individual’s decision alone. Additionally, the monetary saving variable cannot be expressed as a percent of total income due the way this variable and the annual house income variable are constructed in the survey. Nonetheless, annual household income is adjusted for in multivariable analysis. While we adjusted for self-reported health status and excluded participants who were underweight (as a proxy for underlying medical conditions) and with heart disease and stroke, it could still be possible that weight loss occurred as a result of a medical condition. Finally, examining decision making in low-income samples has important health, welfare and policy implications, yet the analytic sample utilized in this study is not representative of the U.S. population at large.

## Conclusions

The present study contributes to the literature by examining the cross-domain behavior of adults, especially from low-income families, exhibiting various levels of LTWL success. More specifically, scant evidence exists focusing on decision-making in the health and financial domain of adults who have been historically overweight and predominately low-income. Thus, it is crucial to find strategies to improve the health and financial decision making of this population, who likely have lower levels of self-regulation [31]. This is particularly important in modern society which encourages consumption of palatable foods (i.e., ‘obesogenic environment’) and goods through social and environment cues [45]. Current study findings indicate that behaviors in two different domains that share the common mechanism of self-regulation are inversely related in this sample. That is, those who achieved the greatest weight loss were significantly less likely to save money. Hence, it might be advisable to address both health behaviors and financial decision making jointly to enhance individuals’ future health and welfare. This finding, however, warrants further longitudinal research in additional samples containing more information pertaining to underlying psychological mechanisms as well as financial behavior.

## Data Availability

The data used for this study is from NHANES, which is publicly available. Data can be downloaded from the U.S. Centers for Disease Control and Prevention website: https://wwwn.cdc.gov/nchs/nhanes/

## Abbreviations

BMI: Body mass index
CI: Confidence interval
LTWL: Long term weight loss
NHANES: National Health and Nutrition Examination Survey
OR: Odds ratio
SE: Standard error
